# Uncovering Research Themes in Simulation‐Based Critical Care Education: A Bibliometric and Cluster Analysis

**DOI:** 10.1111/nicc.70380

**Published:** 2026-03-30

**Authors:** Lang Li, Yuhang Lv, Liang Dong

**Affiliations:** ^1^ Department of Postgraduate Education Taizhou Central Hospital (Taizhou University Hospital) Taizhou China; ^2^ Department of Critical Care Medicine Taizhou Central Hospital (Taizhou University Hospital) Taizhou China

**Keywords:** bibliometric analysis, critical care medicine, global collaboration, research trends, simulation education

## Abstract

**Background:**

Simulation‐based medical education (SBME) has become integral to critical care training, providing a safe and effective platform for clinical skill development, teamwork and patient safety. Despite increasing recognition of its educational value, the global research landscape of SBME in critical care remains insufficiently mapped.

**Aim:**

To conduct a comprehensive bibliometric analysis and elucidate key trends, influential contributors and emerging research themes in SBME for critical care.

**Study Design:**

Literature published between 1981 and 2025 was systematically retrieved from the Web of Science Core Collection. Bibliometric analyses were performed using VOSviewer, CiteSpace and R (version 4.3.3).

**Results:**

A total of 557 eligible publications were identified, revealing a marked increase in research output, with significant peaks in 2015 and 2021. The United States, Canada, the United Kingdom and China were the leading contributors, collectively accounting for the majority of global publications and international collaborations. *Simulation in Healthcare* was the most prolific journal, while *Critical Care Medicine* had the highest citation impact. Harvard University and Johns Hopkins University emerged as the top institutions and William C. McGaghie, Eric R. Cohen and David B. Wayne were among the most influential authors. Keyword cluster analysis revealed five principal thematic areas: (1) Simulation and Outcomes in Acute Care, (2) Competence and Curriculum Development, (3) Patient Safety and Teamwork, (4) Skill Acquisition and Clinical Training and (5) Education, Confidence and Perceptions. Citation burst analysis highlighted ‘simulation’, ‘high‐fidelity simulation’ and ‘cardiopulmonary resuscitation’ as current and emerging research foci.

**Conclusions:**

SBME research in critical care medicine has experienced substantial global growth, with a clear shift toward high‐fidelity, outcome‐driven and technology‐enhanced educational strategies. This bibliometric analysis provides a structured overview of the field's evolution, interdisciplinary scope and future directions.

**Relevance to Clinical Practice:**

Ongoing research should prioritise the evaluation of advanced simulation modalities, real‐world clinical outcomes and underrepresented critical care scenarios to further enhance training efficacy, patient safety and quality of care.

## Impact Statements

- What is known about this topic?
  ○ Simulation‐based medical education (SBME) is a well‐established tool in critical care training, providing a safe and controlled environment for skill development.
  ○ Prior research demonstrates SBME's effectiveness in improving clinical competence, teamwork and patient safety.
  ○ The field lacks a comprehensive and up‐to‐date bibliometric analysis to map its research landscape, trends and emerging areas of focus.
- What this paper adds?
  ○ A systematic bibliometric analysis of 557 publications identifying key contributors, institutions, journals and citation trends in SBME for critical care.
  ○ Five major research themes, including simulation and outcomes in acute care, competence and curriculum development, patient safety and teamwork, skill acquisition and clinical training and education, confidence and perceptions, with recent trends such as high‐fidelity simulation and quality improvement highlighted.
  ○ Evidence‐based guidance for future research, emphasising the evaluation of advanced simulation modalities, real‐world clinical outcomes and expanding SBME to underrepresented critical care scenarios to enhance training efficacy and patient safety.

## 1 Introduction

Critical care is characterised by high‐stakes, complex and time‐sensitive clinical scenarios that require healthcare professionals to make rapid and accurate decisions [1]. Simulation‐based medical education (SBME) has emerged as an essential pedagogical tool, offering a safe and controlled environment in which practitioners can refine their clinical skills and decision‐making capabilities [2]. Unlike traditional educational models, such as the master‐apprentice approach, SBME enables deliberate practice without risk to patients and provides immediate, standardised feedback to learners [3, 4].

## 2 Background / Justification for Bibliometric Analysis

Simulation‐based training (SBT)—a core element of SBME—has transformed education across multiple medical specialties, including critical care, by providing a safe and effective environment for clinical skill development and teamwork [5, 6]. The unpredictable and high‐pressure nature of critical care requires clinicians to possess diverse competencies, both technical and nontechnical, such as communication and rapid decision‐making [7, 8, 9]. A growing body of literature supports the effectiveness of SBT in enhancing educational outcomes, shortening training periods and improving patient safety [3, 4, 5, 10, 11]. SBME offers a structured and standardised framework for training, enabling customisation of learning experiences for learners at different stages [11]. Low‐fidelity simulations are useful for basic skills, while high‐fidelity simulations replicate complex scenarios for advanced practitioners [12]. This risk‐free setting promotes both technical proficiency and critical thinking, fostering higher clinical competence than traditional methods [13].

SBME also facilitates standardisation of training protocols and objective assessment of skill acquisition, which are often difficult to achieve through conventional apprenticeship models [13, 14]. In critical care, SBME has rapidly evolved to utilise high‐fidelity mannequins, virtual reality and task trainers for specific procedures, each serving distinct educational purposes [9, 15, 16]. Immediate feedback further supports continuous skill refinement and educational innovation [17]. However, widespread implementation is challenged by the high cost of equipment, need for trained faculty and lack of standardised competency assessment tools [14]. Additional research is needed to evaluate its long‐term impact and address these barriers.

## 3 Design and Aims

Bibliometrics provides a quantitative and qualitative approach for analysing scholarly literature, offering insights into research trends, collaboration networks and the contributions of key stakeholders within a specific domain [18, 19]. While recent bibliometric analyses have explored simulation in general medical education, few focus specifically on SBME in critical care. Existing studies often lack detailed analysis of the unique requirements and challenges in critical care settings or do not address the impact of advanced simulation technologies on clinical outcomes [20, 21, 22].

Therefore, this bibliometric analysis aims to address these gaps by conducting a comprehensive bibliometric analysis of SBME in critical care medicine. We seek to elucidate the evolution, key trends and academic impact of SBME in this specialty, identify core research areas and emerging themes and contribute to the ongoing advancement of simulation‐based education in critical care.

## 4 Methods

### 4.1 Search Strategy and Data Collection

To conduct a comprehensive bibliometric analysis of SBME in critical care medicine, a systematic literature search was performed using the Web of Science Core Collection (WoSCC). To ensure consistency and accuracy, all searches were conducted on a single day, August 29, 2025. The search encompassed publications from January 1, 1981—the earliest year of relevant literature—to July 10, 2024. The search strategy was formulated as follows [23, 24]: (TS = (‘critic* car*’ OR ‘intensi* car*’ OR ‘critic* ill*’)) AND TS = (‘simulation teaching’ OR ‘simulation training’ OR ‘simulation learning’ OR ‘simulation education’ OR ‘high fidelity simulation’ OR ‘low fidelity simulation’ OR ‘simulation‐based teaching’ OR ‘simulation‐based education’ OR ‘simulation‐based training’ OR ‘simulation‐based learning’ OR ‘simulation‐based medical education’).

The retrieved data included bibliographic details such as the number of publications and citations, titles, author information, institutional affiliations, countries/regions, keywords and journal titles. All records were exported in text format as ‘Full Records and References’ to facilitate subsequent bibliometric analysis. Only English‐language publications were included.

### 4.2 Statistical Analysis

The bibliometric analysis was conducted using Microsoft Excel 2019 (Microsoft Corporation, Redmond, WA, USA), VOSviewer (version 1.6.20), CiteSpace (version 6.3.R1) and R version 4.3.3. All R‐based analyses were performed using the ‘bibliometrix’ package and its web interface ‘biblioshiny’ to ensure reproducibility. Microsoft Excel was employed to compute key bibliometric indicators, including annual publication volume, citation frequency, average citation frequency, journal titles, journal impact factors, publishing countries/regions, institutions and authors.

VOSviewer was used to map institutional collaborations, co‐authorship networks, citation patterns and co‐citation relationships [25]. This tool enabled visualisation and exploration of complex academic interconnections among authors, institutions and publications. Co‐occurrence analysis conducted via VOSviewer illustrated the relationships among keywords. In these visualisations, node size represented the number of publications, line thickness denoted the strength of the link between nodes and node colour indicated cluster affiliation. Link strength was defined by the number of connections an item had with other items [26].

To identify emerging trends and research hotspots, CiteSpace was employed for burst keyword analysis. The parameters were configured as follows: time slicing from January 1981 to August 2025, with 1‐year intervals; node type: keywords; selection criteria: top 5 keywords per segment; pruning method: pathfinder combined with pruning merged networks. Based on these parameters, visual analyses were performed to generate a keyword timeline chart for the research domain of ‘simulation teaching and critical care medicine’. Additionally, R version 4.3.3 was utilised to visualise international collaborations.

The H‐index was applied to quantify both the productivity and citation impact of individual researchers and journals. In this analysis, each author's H‐index was retrieved directly from the WoSCC database to evaluate scholarly contributions in SBME in critical care medicine [27, 28]. Furthermore, the G‐index, which assigns greater weight to highly cited articles and the M‐index, defined as the H‐index divided by the number of years since the author's first publication, were used to assess academic influence and predict future research productivity [27, 29]. Journal Impact Factors (IFs) for 2024 were obtained from the Journal Citation Reports (JCR) and journal categories were based on the broader category as classified in JCR [30]. Journals were also categorised into quartiles (Q1–Q4) according to their field‐specific impact factor rankings to help researchers identify high‐impact publication venues [31].

## 5 Results

### 5.1 General Trends and Information

**FIGURE 1:**
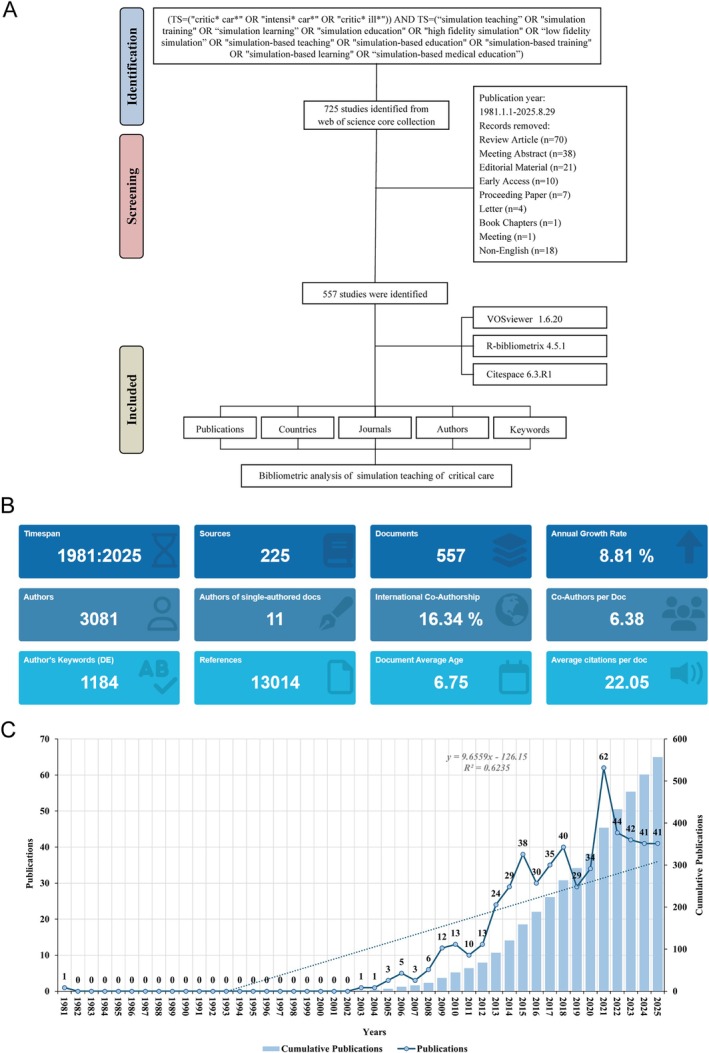
Overview of data screening and publication trends. (A) Flowchart of the data screening process. (B) Annual overview of total publications. (C) Detailed trend analysis displaying yearly publication volume fluctuations.

A total of 725 studies were initially identified through the WoSCC database. Following a rigorous screening process, 168 records were excluded: 70 review articles, 38 meeting abstracts, 21 editorial materials, 10 early‐access articles, 7 proceedings papers, 4 letters, 1 book chapter, 1 meeting and 18 non‐English publications. Ultimately, 557 eligible publications were included in the final bibliometric analysis. The detailed selection process is outlined in Figure 1A.

Descriptive analysis for the 557 included documents is summarised in Figure 1B. These studies were published across 225 sources, authored by 3081 individuals and referencing a total of 13 014 citations. The annual growth rate of publications was 8.81%, with an average of 6.38 co‐authors per document and an international co‐authorship rate of 16.34%, underscoring the collaborative nature of research in this area. On average, each document received 22.05 citations and 1184 unique author keywords were identified, highlighting the breadth of topics covered within SBME in critical care.

From 1981 to 2025, research on SBME in critical care has shown a marked upward trajectory (Figure 1C). The annual number of publications remained low until 2003, after which output steadily increased. Notable milestones included the first substantial rise in 2014 (24 publications) and a significant peak in 2021 with 62 publications. Despite minor fluctuations in subsequent years, annual publication volumes remained robust, with over 40 articles published each year from 2021 onward. The cumulative number of publications has surpassed 550, reflecting sustained and growing scholarly interest in this field.

### 5.2 Analysis of Journals

Analysis of journal distribution revealed the top 20 journals publishing articles on SBME in critical care medicine, as detailed in Table S1. *Simulation in Healthcare—Journal of the Society for Simulation in Healthcare* led in publication volume, with 34 articles (TP rank = 1) and an H‐index of 15, demonstrating its central role in disseminating simulation research. Notably, *Critical Care Medicine* recorded the highest total citation count (TC = 617, TC rank = 1), underscoring its significant academic impact despite a lower publication count (15 articles, TP rank = 4). *Clinical Simulation in Nursing* ranked second in publication volume (29 articles, TP rank = 2) and maintained a solid H‐index of 10, although its total citation count (TC = 250, TC rank = 8) was more modest. Among all journals, *Critical Care Medicine* (IF 2024 = 6.0, Q1), *Chest* (IF = 8.6, Q1) and *Academic Medicine* (IF = 5.2, Q1) exhibited the highest IFs, reflecting strong influence and reputation in the broader clinical and educational community.

**FIGURE 2:**
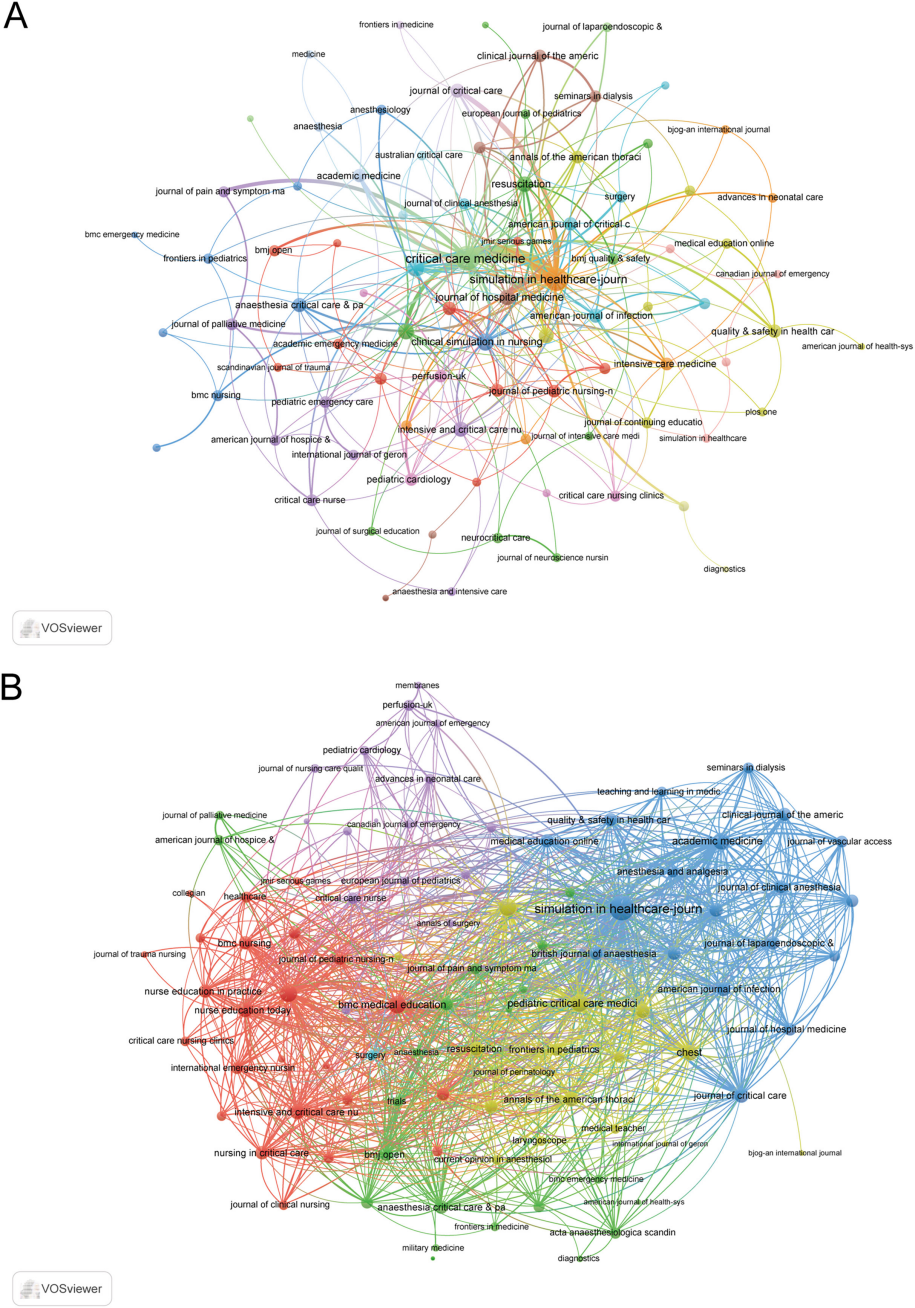
Network analysis of journals. (A) Co‐occurrence networks of journals reflecting thematic or topical connections based on co‐citations. (B) Coupling network of journals, illustrating shared intellectual foundations through common references.

The co‐occurrence network (Figure 2A) demonstrated the three key journals with the highest total link strength were *Critical Care Medicine* (101), *Simulation in Healthcare* (84) and *Clinical Simulation in Nursing* [27]. The journal coupling network further underscored that the top three journals with the highest total link strength were *Simulation in Healthcare* (2842), *Critical Care Medicine* (921) and *Paediatric Critical Care Medicine* (886) (Figure 2B).

### 5.3 Analysis of Countries

**FIGURE 3:**
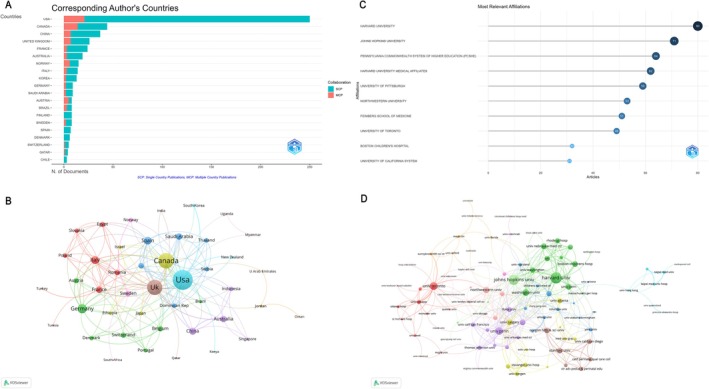
Global and institutional distribution and collaboration. (A) Corresponding authors' publications by country, differentiating between single country publications (SCP) and MCP. (B) Collaboration map among countries. (C) Top 10 institutions by article count and rank.(D) Collaboration map among institutions.

Analysis of the geographic distribution of publications demonstrated that the United States is the clear leader in simulation‐based critical care education research, contributing 250 articles, which represents 44.9% of the total output (Table S2, Figure 3A). Canada and China followed with 44 (7.9%) and 37 (6.6%) publications, respectively. Single Country Publications (SCPs) and Multiple Country Publications (MCPs) were analysed to assess levels of international collaboration. The United States dominated both categories, with 229 SCPs and 21 MCPs, reflecting a strong domestic research base with ongoing, though relatively modest, international partnerships (MCP ratio = 0.084). In contrast, Canada exhibited a much higher MCP ratio (0.318), with 14 of its 44 publications involving international collaborators. Other countries with a notable proportion of MCPs included Norway (MCP ratio = 0.4), Austria (0.625) and the United Kingdom (0.269), suggesting robust global research networks. When considering total citations, the United States again ranked first with 7241 citations, followed by Canada (1194) and the United Kingdom (1053), underscoring the influence and impact of North American and British research. The United Kingdom had the highest average citations per article (40.5), followed by the United States [29] and Canada (27.1), indicating that publications from these countries tend to be highly cited and influential in shaping the field.

In total, 49 countries/regions with a minimum of three co‐authored documents (Figure 3B, Table S3), the United States has the highest total link strength (72), followed by Canada (50) and the United Kingdom (47).

### 5.4 Analysis of Institutions

The top contributing institutions in simulation‐based critical care education research are shown in Figure 3C. Harvard University leads with 80 publications, followed by Johns Hopkins University (71 articles) and the Pennsylvania Commonwealth System of Higher Education (PCSHE) (64 articles). Other major contributors include Harvard University Medical Affiliates (62), University of Pittsburgh (59), Northwestern University (53) and Feinberg School of Medicine (51).

The inter‐institutional collaboration network (Figure 3D) illustrates extensive global partnerships among leading research centres. Among the 96 institutions engaged in international collaborations (with at least three publications), Harvard University demonstrated the highest number of international connections (56), followed by Johns Hopkins University (48) and the University of Toronto [31]. The network map further reveals that prominent US institutions such as Harvard and Johns Hopkins form the core of several robust collaborative clusters, with frequent partnerships spanning North America, Europe and beyond. The University of Toronto, in particular, serves as a central hub for Canadian and international collaborations and Northwestern University, University of Pittsburgh and Mayo Clinic also maintain prominent positions in the global network.

### 5.5 Analysis of Authors

A systematic analysis of author productivity and impact in SBME for critical care medicine identified the most influential contributors in the field: Table S4. William C. McGaghie emerged as the most cited author, with a total of 1827 citations and a leading H‐index of 13. He was closely followed by Eric R. Cohen and David B. Wayne, each with 1820 citations and an identical H‐index of 13. John H. Barsuk ranked fourth, with 1699 citations and an H‐index of 12. In terms of research output, McGaghie led with 15 publications, followed by Cohen and Wayne (each with 14 articles) and Barsuk (13 articles), demonstrating not only productivity but also significant scholarly impact and influence on the field's development.

**FIGURE 4:**
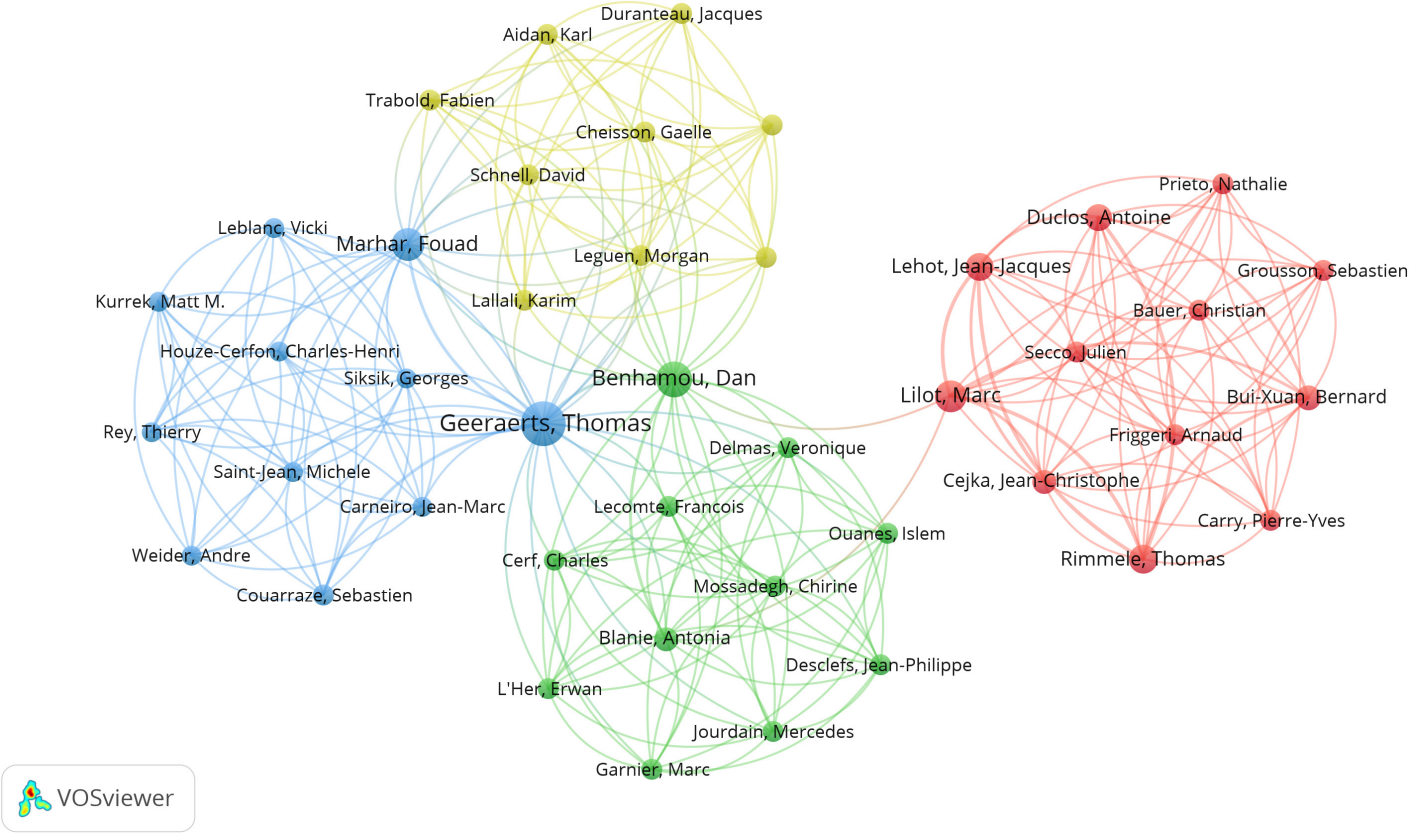
Author Collaboration Network.

Collaboration patterns among leading authors are visualised in Figure 4. Among the 43 authors involved in international collaborations (with a minimum of one publication), Elaine R. Cohen and Diane B. Wayne had the highest number of collaborative links (49 each) [32], followed closely by William C. McGaghie (48 connections) [33].

### 5.6 Analysis of Keywords

**FIGURE 5:**
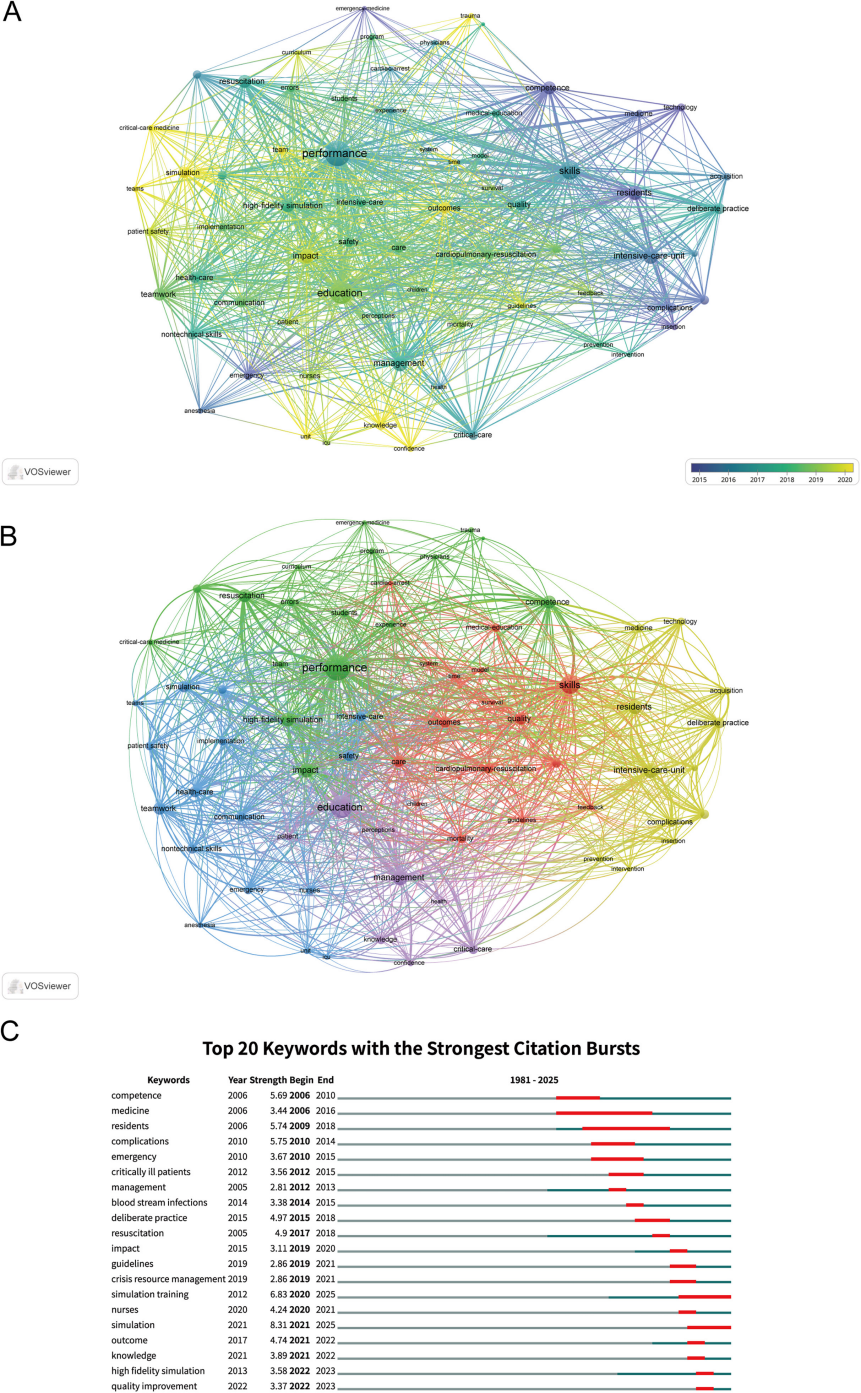
Keyword analysis. (A) Keyword co‐occurrence network. (B) Keyword clusters. (C) Citation burst analysis of keywords.

A total of 70 keywords with at least five occurrences were identified from the literature for co‐occurrence network analysis (Figure 5A). The five most frequently occurring keywords were ‘education’ (96), ‘performance’ (94), ‘skills’ (67), ‘management’ (52) and ‘intensive care unit’ (49), reflecting a central focus on enhancing performance and skills in critical care through structured educational interventions and effective management practices.

Keyword cluster analysis (Figure 5B, Table S5) revealed five thematic clusters. Cluster 1 (red): Simulation and Outcomes in Acute Care. This cluster centres on acute care and resuscitation, featuring terms such as ‘cardiac arrest’, ‘cardiopulmonary resuscitation’, ‘outcomes’, ‘guidelines’, ‘feedback’ and ‘skills’. The theme highlights the role of simulation in improving survival and quality of care in high‐risk, time‐critical situations. Cluster 2 (green): Competence and Curriculum Development. The focus here is on the development of core competencies and structured educational programmes. Keywords include ‘competence’, ‘critical‐care medicine’, ‘curriculum’, ‘high‐fidelity simulation’, ‘performance’, ‘physicians’, ‘students’ and ‘resuscitation’, emphasising the use of simulation to enhance clinical competency and curricular outcomes. Cluster 3 (blue): Patient Safety and Teamwork. This cluster is characterised by a focus on patient safety, communication, teamwork and nontechnical skills in the ICU and healthcare settings. Key terms include ‘adverse events’, ‘patient safety’, ‘teamwork’, ‘communication’, ‘nontechnical skills’, ‘implementation’ and ‘simulation’, highlighting interdisciplinary collaboration and systems‐based approaches to error reduction. Cluster 4 (yellow): Skill Acquisition and Clinical Training. The main theme is the acquisition of practical skills and deliberate practice, especially in procedures and infection prevention. Representative keywords are ‘acquisition’, ‘blood‐stream infections’, ‘complications’, ‘deliberate practice’, ‘intervention’, ‘residents’ and ‘technology’, reflecting simulation's role in hands‐on training and quality improvement. Cluster 5 (purple): Education, Confidence and Perceptions. This cluster addresses broader educational concepts, including ‘confidence’, ‘education’, ‘knowledge’, ‘management’, ‘patient’ and ‘perceptions’, representing the impact of simulation on learner self‐efficacy and attitudes toward critical care.

Analysis of citation bursts (Figure 5C) identified the top 20 keywords that have experienced the most significant surges in scholarly attention, presented in descending order of burst strength and chronology. The strongest citation bursts were observed for ‘simulation’ (strength = 8.31, 2021–2025), ‘simulation training’ (6.83, 2020–2025), ‘complications’ (5.75, 2010–2014) and ‘residents’ (5.74, 2009–2018). Early bursts were seen for ‘competence’ (2006–2010), ‘medicine’ (2006–2016) and ‘residents’ (2009–2018), reflecting foundational interest in skill and knowledge acquisition. More recent bursts—such as for ‘simulation training’ (2020–2025), ‘simulation’ (2021–2025), ‘high fidelity simulation’ (2022–2023) and ‘quality improvement’ (2022–2023)—indicate that these topics have become focal points in the latest research, suggesting future directions in SBME for critical care medicine.

## 6 Discussion

This bibliometric analysis provides a comprehensive bibliometric analysis of SBME in critical care medicine, covering publications from 1981 to 2025. The findings reveal a steady and significant increase in research output, with notable surges in 2015 and 2021. These peaks correspond to key technological milestones, such as the widespread adoption of high‐fidelity simulation and the integration of artificial intelligence (AI) and digital platforms into medical education [34]. The recent acceleration of research activity, especially during and after the COVID‐19 pandemic, further underscores the field's responsiveness to global healthcare challenges and the rising demand for remote and flexible training modalities [35].

It is noteworthy that there were no publications related to simulation‐based critical care education from 1982 to 2002. This absence can be attributed to several factors: the concept of SBME was still in its infancy during those years, with limited access to advanced simulation technology and a lack of formal integration into medical curricula. High‐fidelity simulation, virtual reality and task trainers that are now underpinning SBME only became widely available after the early 2000s. Additionally, academic awareness and research interest in simulation‐based pedagogies for critical care began to increase substantially only as the technology matured and evidence of its effectiveness accumulated.

The United States leads the field by a substantial margin, followed by Canada, the United Kingdom and China, collectively accounting for the majority of SBME research in critical care medicine. Several underlying factors may explain the leadership of the United States: first, strong economic resources and sustained investment in healthcare infrastructure have enabled the rapid adoption of novel simulation approaches and technology in medical education. Second, medical education systems in these countries have prioritised patient safety, competency‐based training and continuous professional development, all of which align closely with the goals of SBME. Third, robust funding programmes from government agencies and private foundations have supported simulation research and implementation, further driving publication output. Similar, though smaller‐scale, trends are observed in Canada and the United Kingdom, where national policies and collaborative research networks have fostered the integration of simulation into critical care training. This dominance reflects both sustained investment in healthcare education and a strong culture of international collaboration, as evidenced by high MCP ratios and robust centrality values for countries like Canada, Austria, Norway and the United Kingdom. These countries have actively prioritised simulation‐based curricula in response to the increasing complexity of critical care and the need for advanced training [14]. The exemplary performance of leading institutions such as Harvard University, Johns Hopkins University and the University of Toronto highlights the role of academic hubs in fostering innovation and global research networks. Their extensive collaborations, as depicted in institutional co‐authorship networks, reinforce the importance of cross‐border partnerships and shared educational priorities [13].

The distribution of research across journals illustrates the interdisciplinary nature of SBME. *Simulation in Healthcare* emerged as the leading publication platform by volume, while *Critical Care Medicine* ranked highest in citation impact. This dichotomy reflects a dual emphasis: simulation‐focused journals drive methodological and pedagogical innovation, whereas broader clinical journals highlight the translational impact of simulation on patient safety and clinical outcomes [15]. The receptiveness of journals such as *Intensive Care Medicine* and *Resuscitation* to SBME research further underscores the integration of simulation approaches into mainstream critical care discourse, bridging the gap between educational theory and clinical practice.

Leading authors—most notably William C. McGaghie, Eric R. Cohen and David B. Wayne—have profoundly shaped the academic landscape of SBME in critical care. Specifically, William C. McGaghie pioneered the concept of mastery learning in medical education, demonstrating that repeated, deliberate practice with feedback can lead to superior clinical skill acquisition and retention. Eric R. Cohen and David B. Wayne have contributed extensively to the development of simulation‐based curricula for critical care procedures and interprofessional team training. Their research has provided robust evidence for the effectiveness of simulation in improving clinical performance, reducing medical errors and enhancing patient outcomes. Furthermore, these authors have developed innovative assessment tools and standardised protocols that have been widely adopted in critical care education. McGaghie's foundational work on mastery learning and deliberate practice has set the standard for structured, competency‐based clinical education [36]. Wayne and Cohen have made substantial contributions to the emphasis on procedural competency, interprofessional teamwork and the application of simulation in high‐stakes environments [33, 37]. The dense network of co‐authorship among these and other influential scholars underscores a thriving culture of scholarly collaboration, which is vital for sustaining methodological rigour and curricular innovation in SBME.

The keyword co‐occurrence network analysis revealed five thematic clusters that reflect the evolving priorities in SBME for critical care.

### 6.1 Cluster 1: Simulation and Outcomes in Acute Care

This cluster centres on acute care interventions and resuscitation, with keywords such as ‘cardiac arrest’, ‘cardiopulmonary resuscitation’, ‘outcomes’, ‘feedback’ and ‘skills’. The co‐occurrence of ‘quality’, ‘model’ and ‘guidelines’ highlights the emphasis on using simulation to drive measurable improvements in patient care and adherence to evidence‐based guidelines. Simulation offers a risk‐free environment for practicing high‐stakes scenarios, ultimately promoting better survival and clinical outcomes. Prior research demonstrates that team‐based simulation training—especially in acute care settings—significantly improves response times and team effectiveness during cardiac arrest, thereby enhancing patient safety [15, 16, 38]. Educational programmes should continue integrating outcome‐focused simulation modules to foster both technical expertise and optimal clinical performance.

### 6.2 Cluster 2: Competence and Curriculum Development

This cluster foregrounds the role of simulation in developing core competencies and structured curricula in critical care. Prominent terms include ‘competence’, ‘critical‐care medicine’, ‘curriculum’, ‘high‐fidelity simulation’, ‘performance’ and ‘students’. The focus on ‘resuscitation’, ‘physicians’ and ‘program’ further emphasises the importance of advanced simulation modalities for clinical skill acquisition. High‐fidelity simulations, combined with deliberate practice, have been shown to outperform traditional training in procedural education by providing repeated, realistic practice opportunities [36, 39]. Learner engagement and feedback mechanisms also emerge as important subthemes, suggesting future curricular strategies should optimise scenario realism, facilitator expertise and structured debriefing.

### 6.3 Cluster 3: Patient Safety and Teamwork

Keywords such as ‘adverse events’, ‘patient safety’, ‘nontechnical skills’, ‘teamwork’ and ‘communication’ underscore the critical importance of simulation for enhancing team‐based care and reducing preventable medical errors. This cluster highlights the vital role of nontechnical skills—such as leadership, decision‐making and communication—in ensuring safe, effective care in the ICU. Simulation‐based team training has been shown to improve communication and coordination, translating to better real‐world outcomes [15, 16, 38]. The inclusion of ‘implementation’ and ‘nurses’ points to the interdisciplinary nature of these interventions. Curricula should therefore include team‐based simulation exercises to promote a culture of safety and collaboration.

### 6.4 Cluster 4: Skill Acquisition and Clinical Training

The focus in this cluster is on procedural skill acquisition and prevention of complications. Keywords such as ‘acquisition’, ‘deliberate practice’, ‘complications’, ‘intervention’, ‘residents’ and ‘simulation‐based education’ reflect the value of simulation in training healthcare professionals to manage common ICU procedures and prevent adverse events. Notably, simulation‐based mastery learning has been shown to reduce iatrogenic complications, such as catheter‐related bloodstream infections [37]. This highlights the need for targeted simulation exercises focused on high‐risk procedures and complication prevention.

### 6.5 Cluster 5: Education, Confidence and Perceptions

This smaller cluster encompasses broader educational concepts, including ‘confidence’, ‘education’, ‘knowledge’, ‘management’ and ‘perceptions’. These terms reflect the impact of simulation on learner self‐efficacy, knowledge acquisition and attitudes toward critical care. The development of confidence and positive perceptions through simulation‐based education is increasingly recognised as essential for preparing healthcare professionals to perform optimally under pressure.

The analysis of citation bursts further revealed emerging and persistent research priorities. Recent years have witnessed strong citation bursts for ‘simulation’ (2021–2025), ‘simulation training’ (2020–2025), ‘high fidelity simulation’ (2022–2023) and ‘quality improvement’ (2022–2023), signalling a growing emphasis on technologically advanced, outcome‐driven and quality‐focused educational strategies. Earlier citation bursts for ‘competence’, ‘medicine’ and ‘residents’ reflect the foundational importance of skill development and clinical training in the literature. Notably, the persistent and recent interest in ‘cardiopulmonary resuscitation’ and ‘high‐fidelity simulation’ [32, 40] underscores the ongoing demand for immersive, realistic training experiences that prepare learners for life‐saving interventions and the complexities of critical care environments.

## 7 Implications for Future Research

The keyword analysis highlights several directions for future research. First, further investigation is warranted into the pedagogical effectiveness of various simulation modalities, particularly ‘high‐fidelity simulation’, in enhancing clinical skills and improving patient outcomes. Second, the strong citation bursts associated with ‘simulation training’ and ‘competence’ underscore the need for continued research into the relationship between simulation‐based education and the development of professional competence in critical care medicine. The citation burst analysis indicates that while certain topics have sustained scholarly interest over time, others have emerged only recently. This trend suggests the necessity of exploring the underlying factors contributing to these variations and anticipating future developments that may influence the evolution of the field.

Furthermore, the analysis reveals potential gaps in the existing literature. For example, although ‘cardiopulmonary resuscitation’ has recently gained increased attention, other critical care scenarios and skill sets may remain underexplored. Future studies should aim to identify these underrepresented areas and examine how simulation education can be effectively tailored to address a broader range of challenges within critical care medicine.

### 7.1 Limitations

Several limitations of this bibliometric analysis must be acknowledged. The reliance on citation counts may not adequately reflect the clinical impact of individual articles, as highly influential work may not necessarily accumulate a large number of citations. Additionally, the exclusion of non‐English publications may introduce a bias favouring English‐speaking research communities, thereby potentially overlooking valuable contributions from non‐English sources. This exclusion may skew the geographic representation of the research landscape, leading to underrepresentation of work from non‐Anglophone regions and limiting the generalizability of our findings to a truly global context.

While this bibliometric analysis provides a comprehensive overview of SBME in critical care, the results are largely descriptive outputs generated from bibliometric software. The bibliometric analysis could be further strengthened by secondary analyses, such as classifying the literature based on specific diseases or techniques related to simulation training and mapping their evolution over time. However, the bibliometric data extracted from the Web of Science Core Collection (WoSCC) database are limited to indexed metadata (e.g., titles, abstracts, author keywords and index terms) and detailed information about specific diseases or simulation techniques is not consistently available. Conducting reliable subgroup or time‐trend analyses based on disease or technology categories would therefore require manual data curation or full‐text mining, which was beyond the scope of the current bibliometric analysis. We have emphasised this limitation and suggest that future research address this issue through manual data classification or advanced text‐mining techniques.

Moreover, the restriction of the literature search to the WoSCC may have introduced selection bias, as relevant publications indexed in other databases such as Scopus or PubMed may have been excluded. Although WoSCC is a reputable and widely used database encompassing a broad range of scholarly literature, future bibliometric studies should consider incorporating multiple databases to ensure a more comprehensive and balanced analysis.

## 8 Conclusion

This comprehensive bibliometric analysis demonstrates robust global growth in SBME research within critical care medicine from 1981 to 2025, marked by significant surges in publication activity in 2015 and 2021. The United States remains the predominant leader, with Canada, the United Kingdom and China also serving as major contributors and hubs for international collaboration. Keyword and cluster analyses revealed five core thematic areas at the forefront of the field: [1] Simulation and Outcomes in Acute Care, [2] Competence and Curriculum Development, [3] Patient Safety and Teamwork, [4] Skill Acquisition and Clinical Training and [5] Education, Confidence and Perceptions. Recent citation burst trends highlight a growing focus on high‐fidelity simulation, cardiopulmonary resuscitation and quality improvement, reflecting the field's shift toward advanced, outcome‐driven and technology‐enhanced educational strategies. Future research should focus on rigorously evaluating the impact of emerging simulation modalities, optimising curriculum integration and expanding simulation‐based approaches to address a broader range of critical care scenarios. Such efforts will be crucial to further enhancing the quality and effectiveness of critical care education and, ultimately, improving patient outcomes.

## Author Contributions

Conception and design: Liang Dong. Administrative support: Liang Dong. Data analysis and interpretation: Lang Li, Yuhang Lv. Manuscript writing: All authors. Final approval of manuscript: All authors.

## Funding

This work was supported by the Natural Science Foundation of China (82072146), Natural Science Foundation of Zhejiang Province (LY21H150003), Medical Science and Technology Project of Zhejiang Province (2021KY401, 2023KY406, 2024KY1815 and 2025HY1440), Science and Technology Plan Project of Taizhou (20ywa25 and 24ywb47), Zhejiang Clinovation Pride (CXTD202501057), Taizhou University Key Medical Research Project (Z2024FSXY03).

## Ethics Statement

This study did not require ethical review approval, as it was based on bibliometric analysis of publicly available data. The research involved no direct interaction with human subjects and did not entail the collection of personal or sensitive information. All data were aggregated from published literature, ensuring that no individuals could be identified or harmed. As a non‐invasive research method, bibliometric analysis focuses solely on trends and patterns in scientific publications, without posing any ethical risks.

## Consent

The authors have nothing to report.

## Conflicts of Interest

The authors declare no conflicts of interest.

## Supporting information


**Table S1:** Bibliometric Indicators of High‐Impact Journals.
**Table S2:** Publication and citation profiles of leading countries.
**Table S3:** Collaboration strength.
**Table S4:** Publication and citation profiles of high‐impact authors.
**Table S5:** Keyword cluster analysis of SBME in critical care medicine.

## Data Availability

All data generated or analyzed during this study are included in this published article.
