# Supplementary material for: Uncovering Research Themes in Simulation‐Based Critical Care Education: A Bibliometric and Cluster Analysis
Source: Nurs Crit Care. 2026 Mar 30;31(3):e70380. doi: 10.1111/nicc.70380 (PMC13111783; doi:10.1111/nicc.70380)
Supplement: Supplementary file 1 — Table S1: Bibliometric Indicators of High‐Impact Journals. Table S2: Publication and citation profiles of leading countries. Table S3: Collaboration strength. Table S4: Publication and citation profiles of high‐impact authors. Table S5: Keyword cluster analysis of SBME in critical care medicine. [file NICC-31-0-s001.docx]

**Table S1 Bibliometric Indicators of High-Impact Journals**

| **Journal** | **H_index** | **G_index** | **M_index** | **IF 2024** | **JCR 2024** | **TP** | **TP_rank** | **TC** | **TC_rank** | **PY_start** |
| --- | --- | --- | --- | --- | --- | --- | --- | --- | --- | --- |
| SIMULATION IN HEALTHCARE-JOURNAL OF THE SOCIETY FOR SIMULATION IN HEALTHCARE | 15 | 29 | 0.938 | 2.1 | Q3 | 34 | 1 | 461 | 2 | 2010 |
| CRITICAL CARE MEDICINE | 12 | 15 | 0.6 | 6.0 | Q1 | 15 | 4 | 617 | 1 | 2006 |
| CLINICAL SIMULATION IN NURSING | 10 | 15 | 0.769 | 2.5 | Q1 | 29 | 2 | 250 | 8 | 2013 |
| PEDIATRIC CRITICAL CARE MEDICINE | 9 | 12 | 0.529 | 4.5 | Q1 | 12 | 5 | 148 | 17 | 2009 |
| BMC MEDICAL EDUCATION | 8 | 11 | 0.8 | 3.2 | Q1 | 18 | 3 | 135 | 20 | 2016 |
| INTENSIVE AND CRITICAL CARE NURSING | 7 | 7 | 0.583 | 4.7 | Q1 | 7 | 12 | 52 | 56 | 2014 |
| NURSE EDUCATION TODAY | 7 | 10 | 0.538 | 4.2 | Q1 | 10 | 6 | 266 | 6 | 2013 |
| RESUSCITATION | 7 | 7 | 0.412 | 4.6 | Q1 | 7 | 15 | 366 | 3 | 2009 |
| CHEST | 6 | 7 | 0.3 | 8.6 | Q1 | 7 | 10 | 205 | 13 | 2006 |
| PEDIATRIC EMERGENCY CARE | 6 | 7 | 0.333 | 1.2 | Q3 | 7 | 14 | 92 | 32 | 2008 |
| AMERICAN JOURNAL OF CRITICAL CARE | 5 | 6 | 0.385 | 2.2 | Q2 | 6 | 16 | 62 | 48 | 2013 |
| AMERICAN JOURNAL OF INFECTION CONTROL | 5 | 5 | 0.417 | 2.4 | Q2 | 5 | 21 | 136 | 19 | 2014 |
| CRITICAL CARE NURSE | 5 | 7 | 0.333 | 2.0 | Q2 | 7 | 11 | 37 | 74 | 2011 |
| FRONTIERS IN PEDIATRICS | 5 | 6 | 0.625 | 2.0 | Q2 | 6 | 19 | 26 | 106 | 2018 |
| JOURNAL OF CRITICAL CARE | 5 | 5 | 0.357 | 2.9 | Q2 | 5 | 25 | 83 | 39 | 2012 |
| JOURNAL OF PEDIATRIC NURSING-NURSING CARE OF CHILDREN & FAMILIES | 5 | 8 | 0.333 | 2.3 | Q1 | 8 | 8 | 26 | 107 | 2011 |
| NURSING IN CRITICAL CARE | 5 | 8 | 0.313 | 2.6 | Q1 | 8 | 9 | 38 | 71 | 2010 |
| ACADEMIC MEDICINE | 4 | 4 | 0.364 | 5.2 | Q1 | 4 | 29 | 353 | 4 | 2015 |
| ANAESTHESIA CRITICAL CARE & PAIN MEDICINE | 4 | 4 | 0.4 | 4.7 | Q1 | 4 | 30 | 18 | 145 | 2016 |
| ANNALS OF THE AMERICAN THORACIC SOCIETY | 4 | 4 | 0.364 | 5.4 | Q1 | 4 | 31 | 24 | 115 | 2015 |

Note(s): H_index: The h-index of the journal, which measures both the productivity and citation impact of the publications. IF: Impact Factor, indicating the average number of citations to recent articles published in the journal. JCR_Quartile: The quartile ranking of the journal in the Journal Citation Reports, indicating the journal's ranking relative to others in the same field (Q1: top 25%, Q2: 25%-50%, Q3: 50%-75%, Q4: bottom 25%). TP: Total Publications. TP_rank: Rank of Total Publications. TC: Total Citations. TC_rank: Rank of Total Citations. Average Citations: The average number of citations per publication. PY_start: Publication Year Start, indicating the year the journal started publication.

**Table S2 Publication and Citation Profiles of Leading Countries**

| **Country** | **Articles** | **Freq** | **SCP** | **MCP** | **MCP_Ratio** | **TP** | **TP_rank** | **TC** | **TC_rank** | **Average Citations** |
| --- | --- | --- | --- | --- | --- | --- | --- | --- | --- | --- |
| USA | 250 | 0.449 | 229 | 21 | 0.084 | 1014 | 1 | 7241 | 1 | 29 |
| CANADA | 44 | 0.079 | 30 | 14 | 0.318 | 189 | 2 | 1194 | 2 | 27.1 |
| CHINA | 37 | 0.066 | 30 | 7 | 0.189 | 149 | 3 | 314 | 5 | 8.5 |
| UK | 26 | 0.047 | 19 | 7 | 0.269 | 102 | 5 | 1053 | 3 | 40.5 |
| FRANCE | 24 | 0.043 | 21 | 3 | 0.125 | 147 | 4 | 430 | 4 | 17.9 |
| AUSTRALIA | 19 | 0.034 | 16 | 3 | 0.158 | 87 | 6 | 252 | 7 | 13.3 |
| NORWAY | 15 | 0.027 | 9 | 6 | 0.4 | 69 | 7 | 261 | 6 | 17.4 |
| ITALY | 14 | 0.025 | 11 | 3 | 0.214 | 60 | 8 | 213 | 8 | 15.2 |
| KOREA | 13 | 0.023 | 11 | 2 | 0.154 | 28 | 16 | 123 | 11 | 9.5 |
| GERMANY | 9 | 0.016 | 7 | 2 | 0.222 | 46 | 9 | 95 | 14 | 10.6 |
| SAUDI ARABIA | 9 | 0.016 | 7 | 2 | 0.222 | 24 | 18 | 52 | 18 | 5.8 |
| AUSTRIA | 8 | 0.014 | 3 | 5 | 0.625 | 26 | 17 | 110 | 12 | 13.8 |
| BRAZIL | 8 | 0.014 | 5 | 3 | 0.375 | 28 | 15 | 9 | 31 | 1.1 |
| FINLAND | 8 | 0.014 | 8 | 0 | 0 | 39 | 12 | 102 | 13 | 12.8 |
| SWEDEN | 8 | 0.014 | 6 | 2 | 0.25 | 42 | 11 | 39 | 20 | 4.9 |
| SPAIN | 7 | 0.013 | 7 | 0 | 0 | 42 | 10 | 34 | 21 | 4.9 |
| DENMARK | 6 | 0.011 | 6 | 0 | 0 | 31 | 14 | 124 | 10 | 20.7 |
| SWITZERLAND | 5 | 0.009 | 4 | 1 | 0.2 | 16 | 21 | 57 | 16 | 11.4 |
| QATAR | 4 | 0.007 | 3 | 1 | 0.25 | 10 | 28 | 24 | 23 | 6 |
| CHILE | 3 | 0.005 | 3 | 0 | 0 | 23 | 19 | 41 | 19 | 13.7 |

Note(s): Articles: Publications of Corresponding Authors only. Freq: Frequence of Total Publications. MCP_Ratio: Proportion of Multiple Country Publications. TP: Total Publications. TP_rank: Rank of Total Publications. TC: Total Citations. TC_rank: Rank of Total Citations. Average Citations: The average number of citations per publication.

**Table S3 Collaboration Strength**

| **Rank** | **Country** | **documents** | **citations** | **total link strength** |
| --- | --- | --- | --- | --- |
| 1 | USA | 274 | 8259 | 72 |
| 2 | Canada | 62 | 2136 | 50 |
| 3 | UK | 34 | 1236 | 47 |
| 4 | Germany | 14 | 142 | 24 |
| 5 | Italy | 17 | 234 | 18 |
| 6 | Spain | 10 | 60 | 18 |
| 7 | China | 40 | 326 | 15 |
| 8 | Ireland | 4 | 59 | 15 |
| 9 | Australia | 26 | 360 | 14 |
| 10 | France | 28 | 442 | 14 |
| 11 | Saudi Arabia | 11 | 135 | 14 |
| 12 | Netherlands | 4 | 66 | 12 |
| 13 | Belgium | 4 | 46 | 11 |
| 14 | Sweden | 14 | 106 | 11 |
| 15 | Switzerland | 8 | 427 | 11 |
| 16 | Austria | 9 | 113 | 10 |
| 17 | Romania | 4 | 12 | 10 |
| 18 | Denmark | 9 | 185 | 9 |
| 19 | Dominican Rep | 1 | 20 | 9 |
| 20 | Egypt | 2 | 7 | 9 |

**Table S4 Publication and Citation Profiles of High-Impact Authors**

| **Authors** | **h_index** | **g-index** | **m-index** | **PY_start** | **TP** | **TP_Frac** | **TP_rank** | **TC** | **TC_rank** |
| --- | --- | --- | --- | --- | --- | --- | --- | --- | --- |
| Cohen ER | 13 | 14 | 0.813 | 2009 | 14 | 2.34 | 2 | 1673 | 2 |
| Mcgaghie WC | 13 | 15 | 0.813 | 2009 | 15 | 2.67 | 1 | 1677 | 1 |
| Wayne DB | 13 | 14 | 0.813 | 2009 | 14 | 2.34 | 3 | 1673 | 2 |
| Barsuk JH | 12 | 13 | 0.750 | 2009 | 13 | 2.31 | 4 | 1564 | 4 |
| Hunt EA | 7 | 7 | 0.583 | 2013 | 7 | 0.76 | 5 | 272 | 8 |
| Barnato AE | 6 | 6 | 0.353 | 2008 | 6 | 1.05 | 8 | 276 | 7 |
| Ala-Kokko TI | 5 | 5 | 0.455 | 2014 | 5 | 0.83 | 9 | 73 | 13 |
| Arnold RM | 5 | 6 | 0.294 | 2008 | 6 | 1.07 | 7 | 182 | 10 |
| Hebbar KB | 5 | 5 | 0.500 | 2015 | 5 | 1.16 | 10 | 57 | 19 |
| Jansson MM | 5 | 5 | 0.455 | 2014 | 5 | 0.83 | 11 | 73 | 13 |
| Kyngäs HA | 5 | 5 | 0.455 | 2014 | 5 | 0.83 | 12 | 73 | 13 |
| Meriläinen MH | 5 | 5 | 0.455 | 2014 | 5 | 0.83 | 14 | 73 | 13 |
| Mohan D | 5 | 5 | 0.333 | 2010 | 5 | 0.91 | 15 | 214 | 9 |
| Ohtonen PP | 5 | 5 | 0.455 | 2014 | 5 | 0.83 | 16 | 73 | 13 |
| Syrjälä HP | 5 | 5 | 0.455 | 2014 | 5 | 0.83 | 18 | 73 | 13 |
| Eisen LA | 4 | 4 | 0.222 | 2007 | 4 | 0.92 | 20 | 169 | 11 |
| Feinglass J | 4 | 4 | 0.250 | 2009 | 4 | 0.65 | 21 | 703 | 5 |
| Geeraerts T | 4 | 4 | 0.500 | 2017 | 4 | 0.40 | 22 | 45 | 20 |
| Jones J | 4 | 4 | 0.286 | 2011 | 4 | 0.37 | 25 | 132 | 12 |
| Nishisaki A | 4 | 4 | 0.250 | 2009 | 4 | 0.27 | 28 | 392 | 6 |

Note(s): H_index: The h-index of the journal, which measures both the productivity and citation impact of the publications. g_index: The g-index of the journal, which gives more weight to highly-cited articles. m_index: The m-index of the journal, which is the h-index divided by the number of years since the first published paper. TP: Total Publications. TP_rank: Rank of Total Publications. TC: Total Citations. TC_rank: Rank of Total Citations. Average Citations: The average number of citations per publication. PY_start: Publication Year Start, indicating the year the journal started publication.

**Table S5 Keyword Cluster Analysis of SBME in Critical Care Medicine**

| Cluster | Key Themes | Prominent Keywords |
| --- | --- | --- |
| 1 | Simulation and Outcomes in Acute Care | Cardiac-arrest, Cardiopulmonary-resuscitation, Care, Children, Feedback, Guidelines, Medical-education, Model, Mortality, Outcomes, Quality, Retention, Skills, Support, Survival, System, Time |
| 2 | Competence and Curriculum Development | Competence, Critical-care medicine, Critically-ill patients, Curriculum, Emergency-medicine, Errors, Experience, High-fidelity simulation, Impact, Performance, Physicians, Program, Resuscitation, Students, Team, Trauma, Ultrasonography |
| 3 | Patient Safety and Teamwork | Adverse events, Anesthesia, Communication, Emergency, Health-care, ICU, Implementation, Intensive-care, Nontechnical skills, Nurses, Patient safety, Safety, Simulation, Teams, Teamwork, Unit |
| 4 | Skill Acquisition and Clinical Training | Acquisition, Blood-stream infections, Complications, Deliberate practice, Insertion, Intensive-care-unit, Intervention, Medicine, Prevention, Residents, Simulation-based edu, Technology |
| 5 | Education, Confidence, and Perceptions | Confidence, Critical-care, Education, Health, Knowledge, Management, Patient, Perceptions |
